# The Association Between Glycemic Variability and Mortality in Critically Ill Patients: A Multicenter Prospective Observational Study

**DOI:** 10.3390/jcm13226939

**Published:** 2024-11-18

**Authors:** Ömer Emgin, Mehmet Yavuz, Adem Şahin, Murat Güneş, Mustafa Eser, Tunzala Yavuz, Damla Kökalan, Bişar Ergün, Kazım Rollas, Mensure Yılmaz Çakırgöz

**Affiliations:** 1 Department of Intensive Care Unit, Tepecik Training and Research Hospital, Konak, Izmir 35020, Turkey; 2 Department of Intensive Care Unit, Izmir Buca Seyfi Demirsoy Training and Research Hospital, Buca, Izmir 35390, Turkey; 3 Department of Intensive Care Unit, Sabuncuoglu Serafettin Training and Research Hospital, Merkez, Amasya 06520, Turkey; 4 Department of Intensive Care Unit, Gümüşhane State Hospital, Merkez, Gümüşhane 29000, Turkey; 5 Department of Internal Medicine and Critical Care, Tekirdağ City Hospital, Süleymanpaşa, Tekirdağ 59100, Turkey

**Keywords:** coefficient of variation, glycemic variability, intensive care, maximal glycemic difference, mortality

## Abstract

**Background:** Glycemic variability (GV) is a prevalent and significant condition observed in critically ill patients. This study aimed to investigate the relationship between early glycemic variability parameters and 28-day mortality in critically ill patients. **Methods:** A multicenter, prospective, and observational study was performed at five tertiary intensive care units (ICUs) in Turkey. All patients who had more than six blood glucose level (BGL) measures per 24 h were included. The parameters of GV including the SD, MGL, MGD (the difference between the maximal and minimal glucose level), and the CV (the percentage of SD to the MGL) in the first 24 h were recorded. **Results:** A total of 578 eligible patients were enrolled in the study, of whom 43.6% were women. The mean age of the patients was 68.09 ± 16.62 years. Overall mortality was 31.5% (n = 182). The glycemic parameters of the CV, SD, and MGD were significantly higher in the non-survivor group than in the survivor group (*p* = 0.040, 0.006, and 0.002, respectively). The multivariate logistic regression analysis revealed that the CV (OR 1.023; 95% CI 1.004–1.042; *p* = 0.017) was an independent factor that increased mortality. Spearman’s rho correlation analysis revealed a strong (r:0.871) and statistically significant correlation (*p* < 0.001) between the CV and MGD. **Conclusions:** The CV calculated within the first 24 h of ICU admission is independently associated with 28-day mortality. The MGD is correlated with the CV and is maybe a practical tool to predict increased risk of mortality at the bedside. However, further studies are needed to establish the independent association of the MGD with mortality.

## 1. Introduction

Dysglycemia is a condition that comprises hyperglycemia, hypoglycemia, and glycemic variability (GV) [1,2]. Dysglycemia is a common condition in critically ill patients with a multifactorial etiology, particularly with diabetic status and activated stressors such as sepsis [3,4]. The incidence of dysglycemia in intensive care units (ICUs) is high and is associated with a high mortality rate [2,3]. Therefore, guidelines recommend the maintenance of blood glucose levels within the standard range [5].

Hypo- and hyperglycemia are parameters that have been studied in critically ill patients for a long time. However, GV, another dysglycemic parameter, is a relatively new parameter that has gained importance in recent years [6]. Glycemic variability is defined as fluctuations in blood glucose levels, which can be measured using intra-day glucose levels, inter-day glucose levels, and HbA1c levels. Several measurements and indices can be used to determine GV, including the standard deviation (SD), coefficient of variation (CV), glycemic lability index (GLI), and mean amplitude of glucose excursion (MAGE) [6]. In addition, a relatively new parameter, the maximum glycemic difference (MGD), has been studied and proposed as a measure of glycemic variability [7]. However, there is no clear consensus on the ideal method for measuring GV in the ICU [1].

Several retrospective studies have shown an independent association between GV and mortality [4,6]. These findings highlight the need to monitor GV in critically ill patients using the best and most practical method.

Currently, there is insufficient research to determine the most appropriate parameter to describe glycemic variability, including its association with mortality in ICU patients. In this multicenter, prospective, and observational study, we aimed to investigate the relationship between early glycemic variability parameters (SD, CV, MGD, MGL) and 28-day mortality in critically ill patients.

## 2. Materials and Methods

### 2.1. Study Design

This multicenter, prospective, and observational study was conducted at five tertiary ICUs (the mixed medical/surgical ICUs) located in four different cities in Turkey. The objective of this research was to establish a correlation between GV parameters in the first 24 h, specifically the CV, SD, MGD, MGL, and short-term (28-day) mortality in the ICU [8]. The Tepecik Training and Research Hospital Local Ethics Committee approved the study protocol (EC number: 2023/03-45, 5 April 2023). All authors declare that the study was conducted in accordance with the Declaration of Helsinki and followed the ethical standards of the country of origin. We started the enrolment of patients in April 2023 at all centers during the same period. A total of 578 eligible patients were enrolled and data collection was completed by all centers by the end of August 2023. All data for the first 24 h were recorded instantly, and the hospital’s electronic system was used for subsequent data [8].

### 2.2. Patients

**Figure 1:**
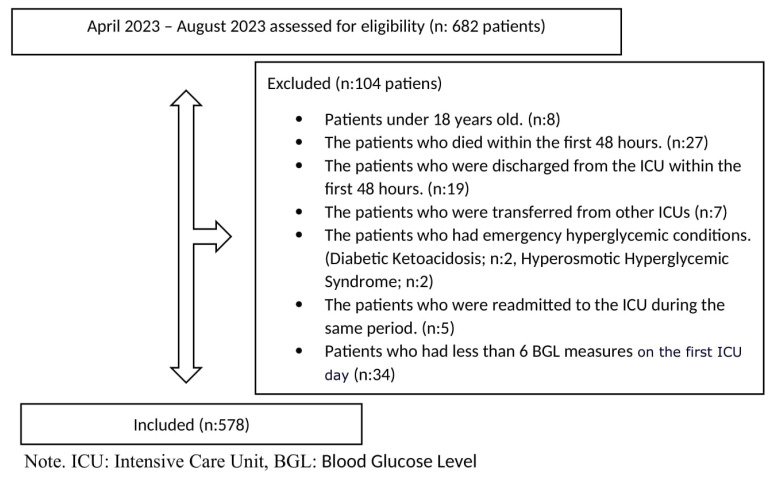
Flowchart of study.

The research analysis included all adult patients aged 18 years or older. Patients who stayed in the ICU for less than 48 h or who had emergency hyperglycemic conditions such as hyperosmotic hyperglycemic syndrome or diabetic ketoacidosis were excluded [7,9,10]. Patients transferred from other ICUs were excluded. All clinicians used the same protocols for the management of glycemia, as recommended by guidelines. We aimed to achieve a BGL of 140 to 180 mg/dL [11,12]. Insulin infusion treatment was initiated if the BGL exceeded 180 mg/dL. If the BGL was below 110 mg/dL after insulin treatment, the infusion treatment was discontinued. If the blood glucose level (BGL) was not within the treatment target, we checked it every 1–2 h. Once the target was achieved, we checked it every 4 h [13]. Bedside fingertip blood glucose measurement (ACCU-CHEK Inform II^®^, Roche Diagnostic, İstanbul, Turkey) was used to evaluate all measures. Patients who had less than 6 BGL measures per 24 h were excluded (Figure 1—flowchart) [13].

### 2.3. Data Collection

Patient characteristics, such as age, gender, underlying diseases, the Acute Physiology and Chronic Health Evaluation II (APACHE-II) score, Sequential Organ Failure Assessment (SOFA) score, the Charlson Comorbidity Index (CCI), the main cause of ICU admission, acute kidney injury (AKI), sepsis/septic shock, length of stay in ICU (LOS-ICU), BGL levels in the first 24 h, biochemical parameters, nutrition type (none, oral, enteral, or total parenteral), insulin therapy, mechanical ventilation support, and 28-day mortality were collected.

The parameters of GV including the SD, MGL, MGD (difference between the maximal and minimal glucose level), and the CV in the first 24 h were recorded [7,8,14]. The coefficient of variation (CV) was calculated as the percentage of the standard deviation (SD) to the mean glucose level (MGL). CV = SD/MGL × 100.

### 2.4. Statistical Analysis

The results of the analysis were presented as percentages, mean ± SD, or medians (interquartile range). The data in the figures were drawn based on the median, interquartile range, and minimum–maximum range. The Chi-squared test was used for categorical variables and the *t*-test was used for normally distributed continuous variables. For non-normally distributed data, the Mann–Whitney U test was used for comparison between groups. Statistical significance was considered for *p*-values ≤ 0.05. Multivariate analysis was performed on the GV parameters of the CV and MGD, which were significant in a univariate analysis. To build the model, a purposeful selection method was used to select a subset of covariates that were considered clinically important, adjusting for confounders and statistical significance [15]. An adjusted odds ratio (OR) and a 95% confidence interval (CI) were reported for each independent factor. Correlation analysis between the CV and MGD was performed using Spearman’s rho. For this analysis, statistical significance was considered at *p*-values < 0.01. The statistical analysis was conducted using SPSS version 26.0 (SPSS Inc., Chicago, IL, USA).

## 3. Results

During the study period, a total of 682 patients were admitted to the ICUs. Out of these, 104 patients were excluded for not meeting the inclusion criteria. Finally, 578 patients who met the inclusion criteria were enrolled for analysis (Figure 1).

The study’s overall mortality rate was 31.5%, with 182 deaths. The mean age of the patients was 68.09 ± 16.62 years, with 252 (43.6%) being female. The main reason for ICU admission was sepsis (41.5%), followed by neurological causes (17.3%), other causes (14.2%), and respiratory causes (13.0%). The study found that the most prevalent comorbidities among the participants were hypertension (HT) at 262 (45.3%), congestive heart disease (CHD) at 167 (28.9%), and diabetes mellitus (DM) at 160 (27.7%). The non-surviving patients had a significantly higher ratio of HT, CHD, and DM compared to the surviving patients (*p* = 0.048, 0.020, and <0.001, respectively) (Table 1).

A comparison of clinical characteristics between survivors and non-survivors is presented in Table 2. The average APACHE-II score for all patients was 23.13 ± 10.70. The non-survivor group exhibited significantly higher APACHE-II scores, SOFA scores, and CCI (*p* < 0.001 for all). The most prevalent method of nutrition was oral 233 (40.3%), followed by enteral, none, and then parenteral. On admission to the ICU, the non-survivor group had significantly higher ratios of vasopressor therapy, insulin therapy, IMV treatment, hemodialysis, and AKI diagnosis (*p* ≤ 0.001, 0.001, <0.001, <0.001, <0.001, respectively). The average duration of IMV for all patients was 6.73 ± 9.03 days. The mean duration of IMV for non-survivors was 9.58 ± 7.84 days, which was significantly higher than for survivors (*p* < 0.001). The mean of the LOS-ICU was 12.22 ± 8.74 days for all patients, and there was no statistically significant difference between the two groups (Table 2).

The results of the statistical analysis for the glycemic variability parameters and HbA1c are shown in Table 3. The median HbA1c in the non-survivor group was 7.60 (6.77–9.32) and significantly higher than in the survivor group (*p* = 0.025). The median CV was 14.45 (1.58–20.74) for survivors and 16.41 (10.83–23.78) for non-survivors (*p* = 0.040). The median SD was 19.78 (14.53–30.74) for survivors and 24.32 (15.42–40.43) for non-survivors (*p* = 0.006). The median MGD was 59.50 (45.00–91.00) for survivors and 74.00 (49.75–125.75) for non-survivors (*p* = 0.002). Glycemic parameters of the CV, SD, MGL, and MGD were significantly higher in the non-survivor group (*p* = 0.040, 0.006, 0.002, respectively). The median MGL was higher in non-survivors compared to survivors. However, the difference was not significant (*p* = 0.056) (Table 3).

The multivariate logistic regression analysis revealed that the APACHE-II score (OR 1.047; 95% CI 1.024–1.069; *p* < 0.001), the CCI (OR 1.212; 95% CI 1.132–1.297; *p* ≤ 0.001), IMV treatment on admission (OR 2.972; 95% CI 1.953–4.521; *p* < 0.001), and the CV (OR 1.023; 95% CI 1.004–1.042; *p* = 0.017) were independent factors that increased mortality (Table 4). We performed another multivariate logistic regression analysis for mortality with the MGD, which was significant in the univariate analysis, using the same variables. We demonstrated that the MGD was not an independent factor for mortality (OR 1.003; 95% CI 1.000–1.006; *p* = 0.056) (Table 5).

**Figure 2:**
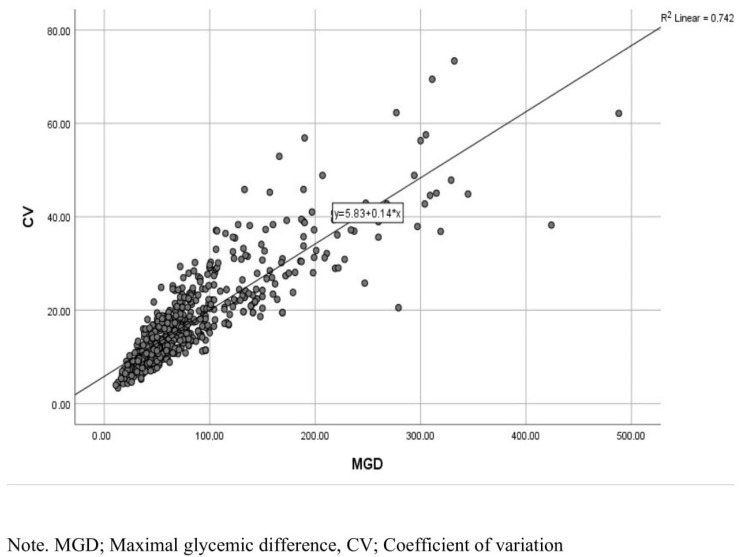
Chart of correlation between maximal glycemic difference and coefficient of variation.

We performed Spearman’s rho correlation analysis between the CV and MGD. There was a strong (r: 0.871) and significant (*p* < 0.001) relationship between the CV and MGD. This study demonstrated that there was a linear positive relationship between two parameters of GV. The results of the analysis are shown in Table 6 and Figure 2.

**Figure 3:**
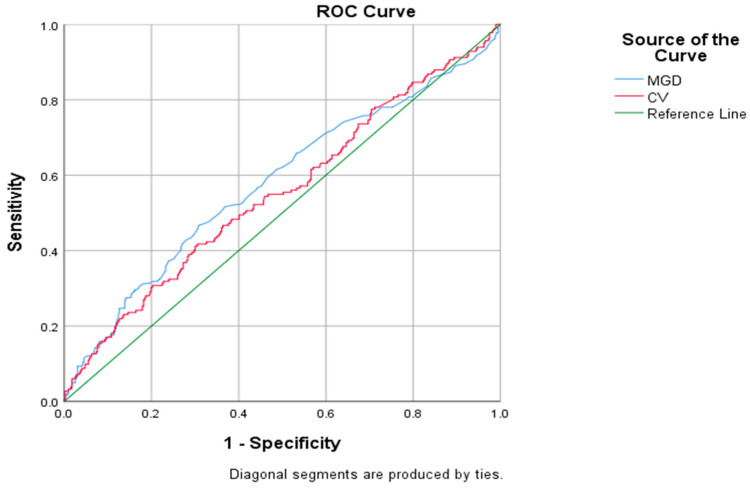
Receiver operating characteristic (ROC) curves to assess the ability of the maximal glycemic difference (MGD) and coefficient of variation (CV).

For the CV and MGD, the adjusted AUCs for detection were 0.553 (95% CI 0.501–0.605) (*p* = 0.004) and 0.578 (95% CI 0.527–0.630) (*p* = 0.002), respectively. A cut-off value of 15.43% was identified as an effective predictor of success for the CV, with a sensitivity of 54.4% and a specificity of 54.0%. Using a cut-off of 65.50 mg/dL was able to predict the success of the MGD with a sensitivity of 56.6% and a specificity of 55.6%. The ROC curves are presented in Figure 3.

## 4. Discussion

The primary objective of this prospective observational multicenter study was to assess the association between GV parameters (MGL, SD, CV, MGD) and 28-day mortality in patients admitted to the ICU. It was found that except for the MGL, all the parameters were associated with short-term mortality in the ICU. Furthermore, the CV was identified as an independent risk factor for 28-day mortality. Although treatment targets for both hypo- and hyperglycemia have been established for a long time [13], it is well documented that fluctuations in glucose levels can negatively impact critically ill patients, similar to the effects of hypo- and hyperglycemia [16]. This has led to the hypothesis that fluctuations in blood glucose levels may have more significant clinical consequences than previously assumed [4]. Our findings align with the existing literature, providing further support for this hypothesis. Additionally, the univariate analysis indicated that the MGD, a relatively new parameter for assessing glycemic variability that can be easily calculated, has a statistically significant effect on mortality.

Excessive glucose fluctuations may lead to increased oxidative stress, hormonal changes, endothelial dysfunction, and activation of pro-inflammatory pathways [2,16]. After demonstrating these negative effects of dysglycemia, numerous clinical studies were conducted to define dysglycemia parameters. In addition to hypoglycemia and hyperglycemia, which are commonly focused on correction, the recognition and treatment of glycemic fluctuations have also become important [14,17]. Researchers have several measures at their command to determine GV, including the MGL, SD, CV, GLI, MAGE, and a relatively new parameter, the MGD [1,7].

Studies examining different parameters in the literature have yielded different results [2]. Hermanides et al. conducted a retrospective study evaluating the mean absolute glucose and the SD and found an association between these parameters and ICU and hospital mortality [18]. In another retrospective study, Kim et al. evaluated the association between 28-day ICU mortality and an early CV measurement as a glycemic variability parameter. They found that an increasing CV was associated with short-term ICU mortality [19]. During the early stages of hospitalization, GV may reflect physiological stress and can therefore be used as a parameter for predicting mortality [20]. Additionally, an increase in glucose fluctuation leads to the activation of oxidative stress, protein kinase C, and adhesion molecules, which in turn induces apoptosis [19,20]. The increased mortality of patients with the SD and CV in this study were in line with the literature and may be explained by pathophysiological underlying mechanisms [19,20]. In the non-survivor group, the MGL was higher, but it did not show a significant difference. This finding may be clarified by the fact that we had a target for blood glucose levels, not for GV parameters, in this study.

The MGD is the range of glucose levels between the maximum and minimum values. This parameter may be calculated at the bedside and more quickly than other parameters [7]. In this study, there was an association between 28-day mortality and the MGD, similar to the literature [7]. A higher MGD indicates a greater fluctuation in glucose levels. This fluctuation could trigger further activation of oxidative stress and other pathophysiological mechanisms [20]. Further randomized controlled studies are needed to investigate the pathophysiological mechanisms of the MGD and the independent effect of the MGD on mortality. The MGD can be calculated at the bedside and more quickly than other GV parameters [7]. Due to the correlation between the CV and MGD revealed in this study, the use of the MGD may be more practical for mortality prediction [14,21]. To the best of our knowledge, there are no studies that search for the correlation between the CV and MGD.

It has long been known that chronic hyperglycemia is detrimental in critically ill patients [20]. Similarly to the literature, it was not surprising that the non-survivor group of this study had a higher HbA1c level, as HBA1c can reflect chronic hyperglycemia [22,23].

Sepsis, a life-threatening syndrome with numerous side effects, is one of the major causes of ICU admission [24]. Similarly, in this study, sepsis was the most common reason for admission and was associated with mortality. At admission, a higher rate of IMV treatment was found to be associated with an increased risk of mortality. It is possible to explain this result by considering that patients who received IMV treatment may have a serious illness, and this treatment can lead to many complications [25]. The most common comorbidity in ICU patients was HT, followed by CHD and DM [26]. After analyzing all our disease severity scores, comorbidities, and reasons for ICU admission, we believe that the results of this study could be generalized in general adult ICU patients.

The mortality rate in this study was 31.5%, which is within the range of the previous trials [20,21]. Various parameters affect mortality in the ICU [22,23]. To clarify the power effect of the CV and MGD on mortality, we conducted multivariate analysis for each parameter separately. In this study, the other parameters that strongly affected mortality, such as age, APACHE II, CCI, and IMV treatment on admission, were used for a multivariate analysis. Furthermore, in studies on mortality in intensive care, these parameters are frequently used in multivariate analyses [7,26,27]. In the multivariate analysis, the CV was found to be an independent factor that significantly increased ICU mortality. Although the MDG was found to increase mortality, its statistical significance was borderline. Similarly to chronic hyperglycemia, early and intermittent hyperglycemia can have harmful effects. These effects include the activation of multiple pro-inflammatory pathways and ischemia [28]. This result may clarify the relationship between early-term mortality and ischemia caused by the CV. The study suggests that the CV may be a target for the treatment of dysglycemia, including hypo- and hyperglycemia, in the ICU. This finding highlights the importance of glucose fluctuation. Therefore, considering glycemic variability may decrease mortality rates. Further research is required to clarify the independent significance of the MGD parameter.

This trial had some limitations. The continuous glucose monitoring system cannot be used. In the same studies, conventional point-of-care blood glucose monitoring has a worse effect on GV and hypoglycemia than continuous glucose monitoring systems [29]. The mortality and comorbidity scores were slightly higher than those reported in other studies [26]. The glycemic parameters selected for this study did not include the MAGE or GLI due to our approach. However, our GV parameters, particularly the CV, were more widely used and applicable in common clinical practice. The major strength of this study was that it was a multicenter prospective study with a large number of ICU patients. We employed a standard glucose target management approach, as recommended by guidelines, in all hospitals with at least one intensive care specialist [13]. All hospitals use the same glucose measurement method with the same machine. By including patients with measurements of six BGLs or more, we aimed to calculate a more accurate GV. In our study, we also included the MGD, which is a very new parameter and easy to calculate.

## 5. Conclusions

The identification of glycemic variability within the first 24 h of ICU admission is critical, as it has been identified as a significant predictor of mortality. The strong association between the coefficient of variation—a commonly used parameter for measuring glycemic variability—and 28-day mortality emphasizes the need to monitor glucose fluctuations beyond just hypo- and hyperglycemia. The maximal glycemic difference, a recently introduced parameter, exhibited a robust correlation with the coefficient of variation, suggesting that it could serve as a practical bedside tool for predicting mortality. These findings highlight the need for research to develop targeted glycemic management strategies in the ICU that take into account glycemic variability, which may serve as an important treatment target.

## Figures and Tables

**Table 1:** Baseline demographic data comparison between survivors and non-survivors.

Baseline Variables	All Patients n: 578 (100.0%)	Survivors n: 396 (68.5%)	Non-Survivors n: 182 (31.5%)	*p*-Value
Age (mean years)	68.09 ± 16.62	66.42 ± 17.17	71.74 ± 14.73	**<0.001**
Gender (n (%))				
Female	252 (43.6%)	183 (46.2%)	69 (37.9%)	0.071
Male	326 (56.4%)	213 (53.8%)	113 (62.1%)	
Comorbidities (n (%))				
Diabetes Mellitus	160 (27.7%)	90 (22.7%)	70 (38.5%)	**<0.001**
Hypertension	262 (45.3%)	168 (42.4%)	94 (51.6%)	**0.048**
Congestive Heart Disease	167 (28.9%)	98 (24.7%)	69 (37.9%)	**0.020**
Coronary Artery Disease	105 (18.2%)	62 (15.7%)	43 (23.6%)	**0.027**
Cerebrovascular Disease	139 (24.0%)	96 (24.2%)	43 (23.6%)	0.917
Chronic Obstructive Pulmonary Disease	132 (22.8%)	90 (22.7%)	42 (23.1%)	0.915
Chronic Kidney Disease	55 (9.5%)	35 (8.8%)	20 (11.0%)	0.446
Liver Disease	13 (2.2%)	6 (1.5%)	7 (3.8%)	0.126
Malignancy	99 (17.0%)	43 (10.9%)	55 (30.2%)	**<0.001**
Causes of ICU Admission (n (%))				
Sepsis	240 (41.5%)	151 (38.1%)	89 (48.9%)	**0.018**
Respiratory Causes	75 (13.0%)	54 (13.6%)	21 (11.5%)	0.594
Neurological Causes	100 (17.3%)	73 (18.4%)	27 (14.8%)	0.344
Trauma	29 (5.0%)	27 (6.8%)	2 (1.1%)	**0.002**
Postoperative Patients	52 (9.0%)	46 (11.6%)	6 (3.3%)	**0.001**
Other Causes	82 (14.2%)	45 (11.4%)	37 (20.3%)	**0.007**

Note: ICUs, intensive care units.

**Table 2:** Comparison of clinical characteristics between survivors and non-survivors.

Clinical Characteristics	All Patients n: 578 (100.0%)	Survivors n: 396 (68.5%)	Non-Survivors n: 182 (31.5%)	*p*-Value
Diagnosis/treatment on admission (n (%))				
Vasopressor therapy (norepinephrine)	187 (32.4%)	75 (18.9%)	112 (58.9%)	**<0.001**
Acute kidney Injury	212 (36.7%)	111 (28.0%)	101 (55.5%)	**<0.001**
Hemodialysis	52 (9.0%)	19 (4.8%)	33 (18.1%)	**<0.001**
Insulin therapy	136 (23.5%)	77 (19.4%)	59 (32.4%)	**0.001**
IMV treatment	237 (41.0%)	121 (30.6%)	116 (63.7%)	**<0.001**
Route of nutrition (n (%)) (first day)				
None	152 (26.3%)	56 (14.1%)	96 (52.7%)	**<0.001**
Oral	233 (40.3%)	194 (49.0%)	39 (21.4%)	**<0.001**
Enteral	134 (23.2%)	103 (26.0%)	31 (17.0%)	**0.019**
Total parenteral nutrition	59 (10.2%)	43 (10.9%)	16 (8.8%)	0.554
Severity scores				
APACHE-II scores	23.13 ± 10.70	20.56 ± 9.30	28.71 ± 11.42	**<0.001**
SOFA scores	6.00 (4.00–8.00)	5.00 (3.00–6.00)	7.00 (6.00–9.25)	**<0.001**
CCI	5.00 (3.00–8.00)	5.00 (3.00–7.00)	7.00 (5.00–10.00)	**<0.001**
Duration of IMV (day)	6.73 ± 9.03	5.42 ± 9.24	9.58 ± 7.84	**<0.001**
Length of stay ICU (day)	12.22 ± 8.74	12.17 ± 9.09	12.34 ± 7.97	0.829

Note: IMV, invasive mechanical ventilation; APACHE-II, Acute Physiologic and Chronic Health Evaluation-II; SOFA, Sequential Organ Failure Assessment; CCI, Charlson Comorbidity Index; ICU, intensive care unit.

**Table 3:** Comparison of glycemic variability parameters/HbA1c among survivors and non-survivors.

Glycemic Variability Parameters	All Patients n: 578 (100%)	Survivors n: 396 (68.5%)	Non-Survivors n: 182 (31.5%)	*p*-Value
* HbA1c (glycated hemoglobin)	6.75 (5.60–8.65)	6.25 (5.45–7.60)	7.60 (6.77–9.32)	**0.023**
Mean glycose level (mg/dL)	146.63 (122.85–172.25)	144.42 (122.35–167.75)	153.15 (124.28–186.05)	0.056
Standard deviation (mg/dL)	20.85 (14.68–32.91)	19.78 (14.53–30.74)	24.32 (15.42–40.43)	**0.006**
Maximal glycemic difference (mg/dL)	64.00 (45.00–98.00)	59.50 (45.00–91.00)	74.00 (49.75–125.75)	**0.002**
Coefficient of variation (%)	14.98 (10.58–21.74)	14.45 (10.58–20.74)	16.41 (10.83–23.78)	**0.040**

Note: * A total of 64 (n: 64) patients had HbA1c levels in the three months prior to their admission.

**Table 4:** Multivariate logistic regression analysis for risk factors for mortality.

Risk Factors	OR (95% CI)	*p*-Value
Age, years	1.002 (0.988–1.017)	0.752
Gender	1.360 (0.895–2.067)	0.150
APACHE-II score	1.047 (1.024–1.069)	**<0.001**
CCI	1.212 (1.132–1.297)	**<0.001**
IMV treatment on admission	2.972 (1.953–4.521)	**<0.001**
Coefficient of variation (%)	1.023 (1.004–1.042)	**0.017**

Note: IMV, invasive mechanical ventilation; APACHE-II, Acute Physiologic and Chronic Health Evaluation-II; CCI, Charlson Comorbidity Index; OR, odds ratio; CI, confidence interval.

**Table 5:** Multivariate logistic regression analysis for risk factors for mortality.

Risk Factors	OR (95% CI)	*p*-Value
Age, years	1.003 (0.988–1.018)	0.689
Gender	1.371 (0.903–2.082)	0.138
APACHE-II score	1.046 (1.024–1.069)	**<0.001**
CCI	1.205 (1.126–1.290)	**<0.001**
IMV treatment on admission	2.951 (1.942–4.483)	**<0.001**
Maximal glycemic difference (mg/dL)	1.003 (1.000–1.006)	0.056

Note: IMV; invasive mechanical ventilation; APACHE-II; Acute Physiologic and Chronic Health Evaluation-II; CCI; Charlson Comorbidity Index; OR, odds ratio; CI, confidence interval.

**Table 6:** Correlation between maximum glycemic difference and coefficient of variation.

MGD		CV
	Spearman’s rho Correlation Coefficient	0.871 *
	*p*	<0.001

Note: * Correlation is significant at the 0.01 level. MDG, maximal glycemic difference; CV, coefficient of variation.

## Data Availability

Data is available upon request to the corresponding author. It is not publicly available due to confidentiality reasons.
